# Deep learning in estimating prevalence and systemic risk factors for diabetic retinopathy: a multi-ethnic study

**DOI:** 10.1038/s41746-019-0097-x

**Published:** 2019-04-10

**Authors:** Daniel S. W. Ting, Carol Y. Cheung, Quang Nguyen, Charumathi Sabanayagam, Gilbert Lim, Zhan Wei Lim, Gavin S. W. Tan, Yu Qiang Soh, Leopold Schmetterer, Ya Xing Wang, Jost B. Jonas, Rohit Varma, Mong Li Lee, Wynne Hsu, Ecosse Lamoureux, Ching-Yu Cheng, Tien Yin Wong

**Affiliations:** 1 Singapore National Eye Center, Singapore Eye Research Institute, Singapore, Singapore; 2 Duke-NUS Medical School, National University of Singapore, Singapore, Singapore; 3 Department of Ophthalmology and Visual Sciences, The Chinese University of Hong Kong, Hong Kong SAR, China; 4 National University of Singapore, School of Computing, Singapore, Singapore; 5 Department of Ophthalmology, Lee Kong Chian School of Medicine, Nanyang Technological University, Singapore, Singapore; 6 Department of Clinical Pharmacology, Medical University of Vienna, Vienna, Austria; 7 Centre for Medical Physics and Biomedical Engineering, Medical University of Vienna, Vienna, Austria; 8 Beijing Key Laboratory of Ophthalmology and Visual Sciences, Beijing Institute of Ophthalmology, Beijing Tongren Eye Center, Beijing Tongren Hospital, Capital Medical University, Beijing, China; 9 Department of Ophthalmology, Medical Faculty Mannheim of the Ruprecht-Karls-University, Mannheim, Germany; 10 University of Southern California Gayle and Edward Roski Eye Institute, Los Angeles, CA USA

**Keywords:** Epidemiology, Risk factors

## Abstract

In any community, the key to understanding the burden of a specific condition is to conduct an epidemiological study. The deep learning system (DLS) recently showed promising diagnostic performance for diabetic retinopathy (DR). This study aims to use DLS as the grading tool, instead of human assessors, to determine the prevalence and the systemic cardiovascular risk factors for DR on fundus photographs, in patients with diabetes. This is a multi-ethnic (5 races), multi-site (8 datasets from Singapore, USA, Hong Kong, China and Australia), cross-sectional study involving 18,912 patients (*n* = 93,293 images). We compared these results and the time taken for DR assessment by DLS versus 17 human assessors – 10 retinal specialists/ophthalmologists and 7 professional graders). The estimation of DR prevalence between DLS and human assessors is comparable for any DR, referable DR and vision–threatening DR (VTDR) (Human assessors: 15.9, 6.5% and 4.1%; DLS: 16.1%, 6.4%, 3.7%). Both assessment methods identified similar risk factors (with comparable AUCs), including younger age, longer diabetes duration, increased HbA1c and systolic blood pressure, for any DR, referable DR and VTDR (*p* > 0.05). The total time taken for DLS to evaluate DR from 93,293 fundus photographs was ~1 month compared to 2 years for human assessors. In conclusion, the prevalence and systemic risk factors for DR in multi-ethnic population could be determined accurately using a DLS, in significantly less time than human assessors. This study highlights the potential use of AI for future epidemiology or clinical trials for DR grading in the global communities.

## Introduction

By 2040, nearly 600 million people will have diabetes worldwide.^1^ Diabetic retinopathy (DR), a major microvascular complication, is a leading cause of vision impairment.^2–4^ Among people with diabetes, about a third have signs of DR, and up to 10% have more severe levels that require referral (referable DR) or are vision-threatening DR (VTDR).^5^ Clinical trials have shown that controlling major risk factors such as hyperglycemia and hypertension can reduce the risk of DR progression.^6–8^ Thus, vision loss from DR can be reduced by 50% or more by screening, appropriate referral and treatment.^9–12^

Despite these important concepts, there is a lack of understanding of the burden of DR, and thus lack of guidance, priority and resources allocated to tackle this in many countries.^13^ Epidemiological studies show substantial variation in the prevalence of DR (e.g., 40% in the U.S., 31% in Africa, and 17.6% in India),^3,14^ and some studies have not been able to confirm the importance of risk factor such as hypertension as a modifiable risk factor.^15^

In many countries, epidemiological studies are critical to document the burden of DR,^16^ and to identify the specific role of modifiable risk factors.^3,8,17,18^ The assessment of DR in such studies, however, has typically relied on an accurate evaluation of retinal photographic images. Such an assessment requires significant resources, including trained manpower, time, and infrastructure. As a result, many countries and regions do not have accurate epidemiological data on DR to establish local strategies and guidelines.^19^

Deep learning system (DLS) an artificial intelligence (AI)-based machine learning technology.^20,21^ It has revolutionized the computer vision field and achieved substantial jumps in diagnostic performance for image recognition, speech recognition, and natural language processing.^20^ In the technical world, DL has been heavily used in autonomous vehicles,^22^ gaming^23,24^, and numerous smartphone applications. In medicine, this technique has shown promising diagnostic performance, across specialties including ophthalmology (e.g. detection of diabetic retinopathy [DR], glaucoma, and age-related macular degeneration from fundus photographs and optical coherence tomographs),^25–30^ radiology (e.g. detection of tuberculosis from chest X rays, intracranial hemorrhage from computed tomography of the brain),^31–34^ and dermatology (e.g. detection of malignant melanoma from skin photographs)^35^.

For DR, it has shown promising diagnostic performance using retinal images,^21,26,27,30,36,37^ when compared to trained human assessors including ophthalmologists. The performance of DLS is comparable to humans in differentiating referable vs non-referable DR.^26,27,36^ An unanswered question is whether associations between DR (detected by DLS) and risk factors are also similar. Such information will lead to greater acceptance of DLS as a plausible, cost-effective alternative tool compared to traditional human assessment for DR, leading to significant resource savings in epidemiological and clinical studies, including clinical trials.

The objective of this study was to evaluate the ability of the DLS to determine the prevalence and risk factors for DR using a multi-ethnic, multi-site dataset of retinal images from epidemiological and clinical studies of people with diabetes. We compared the performance of the DLS in estimating the prevalence and cardiovascular risk factors of any DR, referable DR and VTDR, as compared to the human assessors. In addition, we estimated the time taken to evaluate the assessment of these outcomes between the two methods.

## Results

### Study population

A total of 18,912 patients (93,293 images) with diabetes were analyzed in this study (Supplementary Figure 1). The participants’ demographics, systemic risk factors, and DR severity levels for the eight datasets are shown in Table 1. The mean values (standard deviation) for age, BMI, diabetes duration, SBP, DBP, HbA1c, total cholesterol, and triglycerides of the 8 cohorts of patients were 62.0 (10.8) years, 27.4 (5.3) kg/m^2^, 9.0 (7.9) years, 134.7 (19.2) mmHg, 73.8 (10.6) mmHg, 7.4% (1.7) and 5.0 (1.7) mmol/L and 2.1 (2.5) mmol/L, respectively.

**Table 1:** Patients’ demographics, risk factors and distribution of diabetic retinopathy of the Singapore Integrated Diabetic Retinopathy Screening Program (SiDRP) between 2014 and 2015 (SiDRP 14–15), Singapore Malay Eye Study (SIMES), Singapore Indian Eye Study (SINDI), Singapore Chinese Eye Study (SCES), Beijing Eye Study (BES), African American Eye Study (AFEDS), Chinese University of Hong Kong (CUHK) and Diabetes Management Project Melbourne (DMP Melb)

Patients’ demographics and vascular risk factors	Overall	SiDRP 14-15	SiMES	SINDI	SCES	BES	AFEDS	CUHK	DMP Melb
	Mean (SD)/number (%)	Mean (SD)/number (%)	Mean (SD)/number (%)	Mean (SD)/number (%)	Mean (SD)/number (%)	Mean (SD)/number (%)	Mean (SD)/number (%)	Mean (SD)/number (%)	Mean (SD)/number (%)
Total number of patients	18,912	14,880	763	1128	484	263	492	314	588
Total number of images	93,293	68,286	3952	6329	5284	429	3383	2199	3431
Patients with ungradable retinal images	1596	1184	90	108	44	45	8	13	104
Total number of patients (deemed gradable by DLS)	17,316	13,696	673	1020	440	218	484	301	484
Total number of eyes (deemed gradable by DLS)	34,349	27,392	1346	2040	880	153	968	602	968
Total number of images (deemed gradable by DLS)	85,902	62,941	3515	5803	4925	378	3359	2131	2850
Age (years)	61.99 (10.77)	61.77 (11.01)	62.06 (9.19)	60.38 (9.82)	63.08 (9.67)	59.89 (9.04)	63.77 (10.45)	64.95 (10.8)	64.27 (11.7)
Gender, female	5577 (47.82)	3892 (48.85)	382 (56.76)	482 (47.25)	196 (44.55)	132 (60.55)	292 (60.33)	150 (49.83)	163 (33.68)
*Ethnicity*									
Chinese	6743 (58.19)	5784 (72.59)	N/A	N/A	440 (100%)	218 (100%)	N/A	301 (100%)	N/A
Indian	1972 (17.02)	952 (11.95)	N/A	1020 (100%)	N/A	N/A	N/A	N/A	N/A
Malay	1643 (14.17)	970 (12.17)	673 (100%)	N/A	N/A	N/A	N/A	N/A	N/A
African American	484 (4.18)	N/A	N/A	N/A	N/A	N/A	484 (100%)	N/A	N/A
Caucasian	484 (4.18)	N/A	N/A	N/A	N/A	N/A	N/A	N/A	484 (100%)
Others	262 (2.26)	262 (3.29)	N/A	N/A	N/A	N/A	N/A	N/A	N/A
*Systemic risk factors*									
BMI (kg/m^2^)	27.41 (5.32)	27.07 (4.92)	27.59 (4.82)	26.84 (4.79)	25.29 (3.78)	27.3 (3.94)	32.42 (7.07)	25.98 (5.1)	30.71 (7.47)
Diabetes duration (years)	9.02 (7.92)	7.3 (5.58)	9.24 (8.43)	10.59 (8.99)	10.44 (9.09)	6.63 (6.72)	11.42 (11.44)	12.73 (9.27)	14.68 (10.7)
Systolic blood pressure (mmHg)	134.65 (19.24)	129.43 (16.39)	153.31 (22.77)	139.97 (19.51)	142.16 (19.49)	137.32 (11.02)	134.59 (19.39)	145.16 (20.48)	139.83 (19.05)
Diastolic blood pressure (mmHg)	73.81 (10.55)	71.04 (10.09)	79.14 (10.95)	77.02 (10.01)	76.32 (8.97)	79.46 (6.07)	78.26 (11.01)	78.46 (10.74)	77.17 (8.91)
HbA1c (%)	7.43 (1.67)	7.22 (1.45)	8.48 (2.04)	7.69 (1.7)	7.55 (1.47)	7.43 (3.35)	7.37 (1.85)	7.38 (1.43)	7.72 (1.42)
Total cholesterol (mmol/L)	4.95 (1.69)	4.47 (0.96)	5.43 (1.26)	4.81 (1.17)	4.89 (1.15)	5.03 (1.03)	9.57 (2.45)	4.28 (0.93)	4.66 (1.33)
HDL cholesterol (mmol/L)	1.39 (1.22)	1.33 (0.36)	1.28 (0.3)	1.04 (0.32)	1.17 (0.34)	1.42 (0.27)	2.87 (0.91)	1.33 (0.4)	1.59 (4.46)
LDL cholesterol (mmol/L)	2.77 (1.16)	2.44 (0.81)	3.3 (1.01)	2.97 (0.94)	2.81 (0.89)	3 (0.85)	5.06 (2.05)	2.31 (0.78)	2.48 (1.07)
Triglycerides (mmol/L)	2.13 (2.51)	1.57 (1.07)	1.8 (1.18)	1.94 (1.2)	1.58 (1.16)	2.01 (1.25)	8.96 (5.62)		1.84 (1.39)
*Diabetic retinopathy distribution by Human assessors* ^a^									
Any DR	2775 (16.03)	1470 (10.73)^b^	233 (34.62)	347 (34.02)	120 (27.27)	15 (6.88)	91 (18.8)	204 (67.77)	295 (60.95)
Referable DR	1098 (6.34)	400 (2.92)^b^	89 (13.22)	102 (10)	42 (9.55)	14 (6.42)	55 (11.36)	156 (51.83)	240 (49.59)
Vision-threatening DR	633 (3.66)	238 (1.74)^b^	41 (6.09)	55 (5.39)	13 (2.95)	10 (4.59)	22 (4.55)	52 (17.28)	202 (41.74)
*Diabetic retinopathy distribution by DLS* ^a^									
Any DR	2737 (15.81)	1405 (10.26)	170 (25.26)	410 (40.2)	152 (34.55)	28 (12.84)	112 (23.14)	183 (60.8)	277 (57.23)
Referable DR	1123 (6.49)	425 (3.1)	103 (15.3)	146 (14.31)	67 (15.23)	12 (5.5)	28 (5.79)	139 (46.18)	203 (41.94)
Vision-threatening DR	698 (4.03)	207 (1.51)	77 (11.44)	87 (8.53)	37 (8.41)	11 (5.05)	12 (2.48)	113 (37.54)	154 (31.82)

Referable diabetic retinopathy (referable DR) was defined as moderate non-proliferative DR (NPDR) or above, including diabetic macular edema (DME)

^a^The grade of the worse eye from each patient was used. If one of two eye is ungradable; the grade of the other eye was taken. If both eyes were ungradable, then the patient was classified as ungradable

^b^For analysis of Singapore Diabetic Retinopathy Screening Program 2014–15 (SiDRP 14–15), DR and DME gradings was based on the available Ophthalmologists’ gradings

### Diagnostic performance

**Fig. 1:**
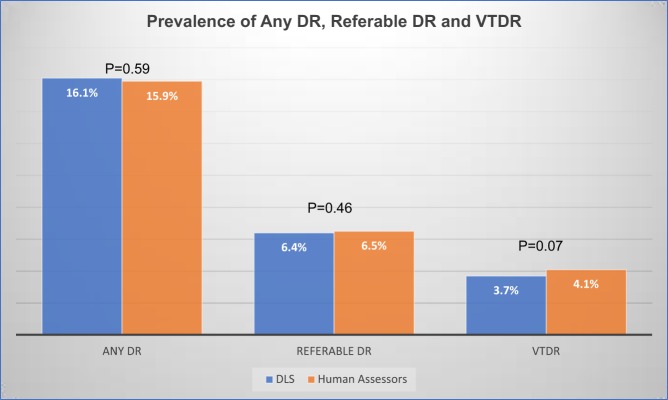
The prevalence of any diabetic retinopathy (DR), referable DR, and vision-threatening DR (VTDR) detected by a deep learning system and human assessors

For the combined pooled dataset, the AUCs of DLS, with reference to the human assessors’ grading, was 0.863 (95%CI: 0.854, 0.871) for any DR, 0.963 (95% CI: 0.956, 0.969) for referable DR, and 0.950 (95% CI: 0.940, 0.959) for VTDR. The overall prevalence of any DR, referable DR, and VTDR was 15.9, 6.5, and 4.1%, respectively, for human assessors vs 16.1, 6.4, and 3.7% for DLS (Fig. 1).

To analyze 93,293 images, the total time taken for a DLS vs human assessor were 10.4 h vs 1554.8 h (Table 2), with the specific details shown in Supplementary Table 1. For the images ‘deemed’ ungradable by the DLS, the additional time required for manual grading was added onto the total time taken. A total of 7391 images ‘deemed’ ungradable by the DLS underwent a secondary manual grading by human assessors, requiring additional 123.2 h (19.0 man-days), totaling up to 125.4 h (21.1 man-day).

**Table 2:** The total number and time taken of retinal images analyzed by a deep learning system (DLS) and a human assessor

	Overall combined dataset^a^	
Patients’ demographics and vascular risk factors	Images (patients)	
Total number of images (patients)	93,293 (18,912)	
Total number of images (deemed gradable by DLS)	85,902 (17,316)	
Ungradable retinal images (patients)	7391 (1596)	
Grading methods	DLS (0.4 s/image)	Human assessors
Time taken to analyze all images (hours)	51.8	3600.1
Time taken to analyze all images (man-days)^b^	2.16	553.9
Additional time taken for secondary manual grading for DLS ungradable images (hours)	123.2	N/A
Additional time taken for secondary manual grading for DLS ungradable images (man-days)^b^	19.0	N/A
Total time taken (man-days)	21.1	553.9
Total time taken (weeks)	4.2	110.7

^a^Overall combined dataset consists of Singapore Integrated Diabetic Retinopathy Screening Program (SiDRP) between 2014 and 2015 (SiDRP 14-15), Singapore Malay Eye Study (SIMES), Singapore Indian Eye Study (SINDI), Singapore Chinese Eye Study (SCES), Beijing Eye Study (BES), African American Eye Study (AFEDS), Chinese University of Hong Kong (CUHK), and Diabetes Management Project Melbourne (DMP Melb). Each image requires 0.4 sec to be analyzed by DLS

^b^1 man-day is equivalent to 6.5 h/day; 5 working days are included in a working week for human. These tables did not include the annual/sick leave or public holidays. The man-day calculation is not applicable to DLS as it can run 24 h a day

Table 3 shows the relationship of risk factors for the DR outcomes evaluated by DLS vs human assessors. Longer duration of diabetes, increased HbA1c and SBP were significantly associated with any DR, referable DR and VTDR (*p* < 0.001) for both DLS and human assessors. Supplementary Table 2 shows the analysis for individual dataset. Combining all datasets, the systemic risk factors were comparable between DLS and human assessors to discriminate any DR (0.738 vs 0.743, *p* = 0.69), referable DR (0.795 vs 0.782, *p* = 0.40), and VTDR (0.810 vs 0.813, *p* = 0.85; Supplementary Figure 2), with the specific AUC of each dataset shown in Supplementary Figure 3.

**Table 3:** The meta-analysis of systemic vascular risk factors with any diabetic retinopathy (DR), referable DR and vision-threatening DR diagnosed by deep learning system, as compared to human assessors in Singapore Integrated Diabetic Retinopathy Screening Program (SiDRP) between 2014 and 2015 (SiDRP 14-15), Singapore Malay Eye Study (SIMES), Singapore Indian Eye Study (SINDI), Singapore Chinese Eye Study (SCES), Beijing Eye Study (BES), African American Eye Study (AFEDS), Chinese University of Hong Kong (CUHK), and Diabetes Management Project Melbourne (DMP Melb)

	Meta-analysis (*n* = 17,316)														
	Any DR					Referable DR					Vision-threatening DR				
	DLS (OR, 95% CI)*	*P* value*	Human (OR, 95% CI)*	*P* value*	*P* value**	DLS (OR, 95% CI)*	*P* value*	Human (OR, 95% CI)*	*P* value*	*P* value**	DLS (OR, 95% CI)*	*P* value*	Human (OR, 95% CI)*	*P* value*	*P* value**
Age (years)	0.98 (0.82, 1.19)	0.87	0.76 (0.7, 0.84)	<0.001	0.018	0.67 (0.59, 0.76)	<0.001	0.66 (0.58, 0.76)	<0.001	0.94	0.62 (0.53, 0.72)	<0.001	0.68 (0.58, 0.8)	<0.001	0.40
Gender (female)	0.93 (0.61, 1.42)	0.73	0.89 (0.67, 1.17)	0.40	0.86	0.88 (0.54, 1.45)	0.62	0.79 (0.52, 1.2)	0.28	0.75	0.74 (0.47, 1.16)	0.19	0.88 (0.54, 1.42)	0.596	0.61
Duration of diabetes (years)	1.43 (1.22, 1.68)	<0.001	1.53 (1.23, 1.9)	<0.001	0.64	1.48 (1.15, 1.89)	0.002	1.4 (1.11, 1.78)	0.005	0.77	1.32 (1.01, 1.73)	0.043	1.41 (1.03, 1.92)	0.031	0.76
HbA1c (%)	1.61 (1.45, 1.79)	<0.001	1.55 (1.44, 1.67)	<0.001	0.55	1.74 (1.54, 1.95)	<0.001	1.74 (1.51, 1.99)	<0.001	0.99	1.58 (1.42, 1.77)	<0.001	1.65 (1.37, 1.97)	<0.001	0.72
Systolic blood pressure (mmHg)	1.54 (1.25, 1.91)	<0.001	1.57 (1.34, 1.83)	<0.001	0.92	1.73 (1.43, 2.09)	<0.001	1.8 (1.39, 2.33)	<0.001	0.82	1.94 (1.52, 2.48)	<0.001	1.71 (1.2, 2.43)	0.003	0.56
Diastolic blood pressure (mmHg)	0.78 (0.66, 0.93)	0.005	0.79 (0.7, 0.89)	<0.001	0.94	0.75 (0.63, 0.9)	0.002	0.68 (0.55, 0.86)	0.001	0.53	0.78 (0.62, 0.97)	0.026	0.87 (0.62, 1.2)	0.39	0.58
Body mass index (kg/m^2^)	0.91 (0.75, 1.11)	0.36	0.87 (0.8, 0.95)	0.002	0.69	0.91 (0.75, 1.11)	0.36	0.92 (0.76, 1.11)	0.37	0.98	0.9 (0.75, 1.1)	0.31	0.93 (0.75, 1.15)	0.50	0.86
Total cholesterol (mmol/L)	0.92 (0.83, 1.03)	0.15	0.95 (0.82, 1.1)	0.52	0.72	0.95 (0.84, 1.07)	0.37	0.98 (0.84, 1.16)	0.85	0.70	0.96 (0.83, 1.12)	0.63	1.08 (0.84, 1.37)	0.56	0.45
Triglycerides (mmol/L)	0.93 (0.85, 1.03)	0.15	0.95 (0.87, 1.04)	0.28	0.80	0.94 (0.83, 1.07)	0.37	0.95 (0.83, 1.1)	0.51	0.92	0.91 (0.77, 1.08)	0.30	0.98 (0.83, 1.16)	0.81	0.57

Any DR: defined as mild non-proliferative DR (NPDR) or worse. Referable DR: defined defined as moderate NPDR or worse, including diabetic macular edema. Vision-threatening DR: defined as severe NPDR and proliferative DR

*OR* standardized odd ratio

*P value is generated by meta-analysis of multivariate logistic regression across 8 datasets

**P value for the statistical difference of multivariate meta-ORs between deep learning system and human assessors, generated using Student’s *t*-test (2-tailed)

**Fig. 2:**
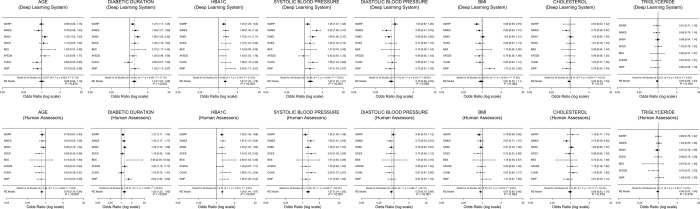
The forest plot of systemic risk factors for any diabetic retinopathy generated by deep learning versus human assessors. These risk factors include age, duration of diabetes, HbA1c, systolic and diastolic blood pressure, body mass index, cholesterol, and triglyceride

**Fig. 3:**
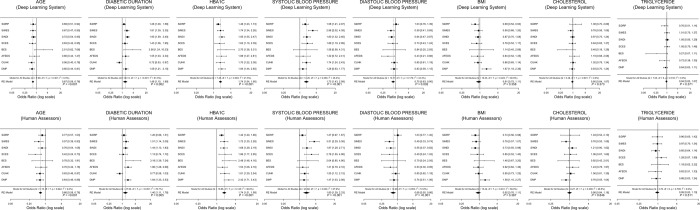
The forest plot of systemic risk factors for referable diabetic retinopathy generated by deep learning versus human assessors. These risk factors include age, duration of diabetes, HbA1c, systolic and diastolic blood pressure, body mass index, cholesterol, and triglyceride

**Fig. 4:**
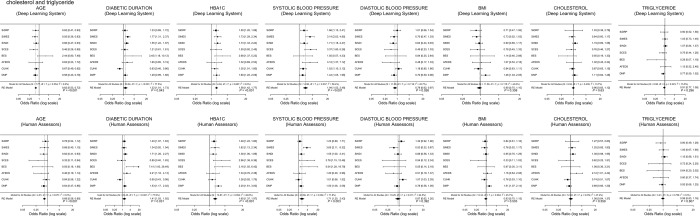
The forest plot of systemic risk factors for vision-threatening diabetic retinopathy generated by deep learning versus human assessors. These risk factors include age, duration of diabetes, HbA1c, systolic and diastolic blood pressure, body mass index, cholesterol, and triglyceride

Using forest plot meta-analysis, both grading methods identified similar risk factors, including younger age, longer diabetes duration, increased HbA1c and systolic blood pressure, for any DR (Fig. 2), referable DR (Fig. 3), and VTDR (Fig. 4). In contrast, gender, total cholesterol, and triglycerides were not associated with DR assessed using both methods.

## Discussion

AI using deep learning techniques may potentially revolutionize how medical images are analyzed.^25,38^ The challenge of AI technology is acceptance by physicians, researchers, and policy makers in terms of robustness and validity of outcomes measured by AI. Besides the obvious potential of using AI in direct clinical care, another immediate application of AI is in research settings, such as in evaluating outcomes in epidemiological studies and clinical trials.

The objective of our study was to evaluate the ability of an AI-based DLS to assess retinal images for DR in population-based epidemiological and hospital-based clinical studies of people with diabetes. We compared results between the DLS and humans in the two key outcomes traditionally measured in such studies (i.e., prevalence and risk factors). We demonstrated comparable outcomes in detecting DR prevalence and risk factor associations between a DLS which was 360 times faster than human assessors. Both the DLS and humans identified a similar prevalence (burden) of DR in the population assessed and longer duration of diabetes, higher HbA1c and higher SBP as risk factors associated with DR. The discriminative ability of these risk factors for DR were comparable between DLS and human assessors. Our study shows while AI technology may need to overcome substantial hurdles, including medico-legal challenges, for application in clinical care,^39,40^ AI technology is an acceptable research tool for assessing outcomes (in this case DR) in population-based and clinical studies, and is particularly suitable for application in countries without the resources to do full-scale research studies.

Our study showed that DLS is a faster grading tool than human assessors, with immediate availability of the outcome. A particular example is SiMES, which is a population-based study conducted in Singapore.^41^ We have previously documented the prevalence and risk factors for this cohort, reporting prevalence of any DR to be 25.5%,^42^ risk factors of longer diabetes duration, higher HbA1c and systolic blood pressure and; protective factors of older age and higher total cholesterol level.^41^ Using the DLS would have resulted in identical findings (Supplementary Table 2). We estimated that in SiMES, the trained human assessor spent ~2–5 min per image, but with DLS, it requires only 0.4 sec.

In total, given that they have a 6.5-man-day (5 days a week), a human assessor would require 553.8 man-days (>2 years) to complete 93,293 retinal images, without factoring the annual/medical leaves and public holidays. In Singapore, the cost for a human assessor, on average, is budgeted to grade about 9800 patients/year. In other words, a human assessor would require about 2 years to grade ~18,000 patients (100,000 retinal images). For DLS, it correspondingly took about 10 h. Even then, for those images deemed ungradable by DLS (~7.9%), these images will need to be graded secondarily by human assessors and hence, additional time (43.5 man-days) was included in our study. On average, the difference between a DLS (with manual grading) vs a human assessor is ~1 month vs 2 years.

Of the risk factors, HbA1c, duration of diabetes and SBP were the most common risk factors associated with increasing DR severity (*p* < 0.001) on the forest plot. These risk factors were consistent with published data from cross-sectional and longitudinal diabetic cohorts.^43–45^ Thus, our study shows the robustness of the DLS as an alternative tool for DR grading and could be utilized to analyze thousands or millions of retinal images over a short period of time. For countries, research institutions, community and hospital health care systems worldwide with limited manpower or financial resources, DLS could potentially save significant time and cost

Our study was limited by the DR grading determined based on mostly 2-field retinal photographs instead of the classic standard 7-field stereoscopic Early Treatment DR Study (ETDRS) field, though 7-field photography would take longer and has higher financial implications. In addition, we also did not have the information on the types of diabetes (e.g. Type 1 vs type 2) of the patients. Future studies could evaluate the generalizability of the DLS for diabetic cohorts with different retinal cameras, settings, and imaging modalities such as ultra-wide retinal photography in detection of DR. It will be important to explore the use of multi-modal machine learning approach in combining the clinical data and retinal images to risk stratify patients with diabetes.

AI-based DLS is a potential alternative assessment tool to determine the epidemiology of DR in research settings, and results in robust, comparable prevalence and systemic risk factors for DR. This technology could potentially transform the conduct of large-scale population-based epidemiological studies, including clinical trials.

## Methods

### Study approval

This study was approved by the Centralized Institutional Review Board (IRB) of SingHealth, Singapore (protocol number SHF/FG648S/2015) and conducted in accordance with the Declaration of Helsinki. Given the retrospective analysis using de-identified images, informed consent was exempted by IRB.

### Development and validation of DLS

The clinical, technical details and diagnostic performance of the DLS have been described previously.^26^ In brief, the DLS was trained using 76,370 retinal images (2-field: optic disc- and macula-centered images), consisting of 88.3% no DR, 6.4% mild non-proliferative DR (NPDR), 3.8% moderate NPDR, and 1.5% VTDR (severe NPDR and proliferative DR). The DR severity level was classified using the International Clinical Diabetic Retinopathy Severity Scale (ICDRSS).^46^ Any DR was defined as mild NPDR (i.e., only microaneurysms) or worse; referable DR as moderate NPDR (i.e., mild NPDR with scattered retinal hemorrhages and hard exudates) or worse; and VTDR as severe NPDR and PDR. If more than one-third of the photo was obscured, it was considered as “ungradable”. All retinal images used to develop the DLS were obtained from diabetes patients attending Singapore National DR Screening Program (SiDRP) from 2010 to 2013.^47^

We have previously validated the DLS,^26^ using 11 separate datasets, with excellent performance, with area under the receiver operating curve (AUC) in detecting referable DR ranging from 0.889 to 0.983.

### Study populations

For this current study, we used 8 multi-ethnic datasets to determine the prevalence and risk factors of DR, including 6 population-based studies: SiDRP with participants from 2014–15,^47^ Singapore Malay Eye Study (SIMES),^42^ Singapore Indian Eye Study (SINDI),^42^ Singapore Chinese Eye Study (SCES),^47^ Beijing Eye Study (BES),^48^ and African American Eye Disease Study (AFEDS),^49^ and two hospital-based studies: Chinese University of Hong Kong (CUHK),^50^ and Diabetes Management Project (DMP), Melbourne.^51^ These 8 datasets had risk factors for DR evaluated using similar definitions and methods. We did not include the other 3 datasets (Guangdong, Mexico and University of Hong Kong) due to the absence of systemic information. We standardized the diagnosis of diabetes as a self-reported history of diabetes, current use of diabetic medications, fasting glucose of ≥7 mmol/L, and/or a non-fasting glucose of 11.1 mmol/L or higher at the time of examination.

Details of the different populations have been described previously. SiDRP was started in 2010 as national DR screening program that covers all public primary eye care hospitals in Singapore via a tele-ophthalmology platform.^26,47^ SiMES, SINDI, and SCES were population-based studies that included participants of three major ethnic groups in Singapore, aged 40–80 years, recruited over an 8-year period: SiMES (Malays, 2004–2006), SINDI (Indians, 2007–2009), and SCES (Chinese, 2009–2011).^42^ The BES was a population-based study in China that involved participants aged 40 years and beyond.^48^ Among these population-based studies, we only included those with diabetes in the analyses. AFEDS is a population-based study of African American aged 40 years and older residing in the city of Inglewood, California. Given that the study was still in active recruitment phase, we only included participants with diabetes recruited up till mid-2017 for this analysis. We included two clinic-based studies among patients with diabetes: the CUHK study was a clinic-based cohort for patients with diabetes, recruited in 2016 from a tertiary eye clinic in Hong Kong,^50^ and the DMP is a clinical-based cohort of patients with diabetes in an eye hospital in Melbourne, Australia.^51^

### Retinal images and DR classification

During DLS training, the input to the neural network was a retinal image, and the individual DR severity levels (0, 1, 2, 3, and 4 for no DR, mild NPDR, moderate NPDR, severe NPDR, and PDR respectively, using ICDRSS classification) were represented by output nodes. The weights of the DLS were adjusted with stochastic gradient descent, to train a classification model for DR. During validation, the DLS model predicted a raw confidence score for each severity level output node, for each image. These node scores were finally linearly weighted to produce a single image-level DR score. An ensemble of two separate models – one trained with the original image, and one with its contest-equalized version – was used. DLS hyperparameters and score thresholds were selected using a set of held-out images.

During validation, for each eye of each patient, the ensembled DR scores of all valid retinal images were averaged to produce an eye-level DR score, for each DR severity level. The predicted DR grade was then obtained by applying the previously-specified score thresholds. For each patient, the grade of the eye with the most severe DR as predicted was used to assess the relationship with systemic risk factors. If one of the two eyes was ungradable, the grade of the other was taken. If both eyes were ungradable, then the patient was classified as ungradable and excluded from the DLS analysis. Based on the training set, we pre-set the optimal operating threshold for any DR, referable DR and VTDR. Ungradable images and eyes with previous retinal laser were not included as part of the analyses.

### Retinal photography protocol, classification, and grading of retinal images

All participants in the 8 datasets underwent 2-field (optic disc- and macula-centered) retinal photography. SiDRP, AFEDS, DMP, and CUHK cohorts were imaged using Topcon retinal camera (Tokyo, Japan) while SiMES, SINDI, SCES, and BES used a Canon retinal camera (Tokyo, Japan).^42,47–51^ For SiDRP, SiMES, SiNDI, SCES, AFEDS, and DMP Melbourne, the images were assessed by the human assessors who were non-ophthalmologists.^42,47,49–51^ The human assessors for BES were a board-certified ophthalmologist and a retinal specialist while CUHK patients were examined by 2 retinal specialists.^48^

### Assessment of systemic risk factors

All datasets consisted of comprehensive patients’ demographics and systemic risk factors (e.g. age, gender, ethnicity, duration of diabetes, HbA1c, systolic and diastolic blood pressure [SBP and DBP], body mass index (BMI), total cholesterol, and triglyceride levels).

### Assessment of time taken for image analysis

The grading time of each retinal image was obtained from the individual study center. The SiDRP, AFEDS, and DMP images were graded at the Singapore Eye Research Institute (SERI) and the SiMES, SINDI, and SCES photos at the Blue Mountain Eye Study reading center in Sydney, Australia. Beijing and Hong Kong cohorts were graded by the ophthalmologist and retinal specialists respectively. The average time taken per image for SiDRP assessors was 2 minutes; CUHK: 5 min; and the remaining (SIMES, SINDI, SCES, BES, AFEDS, and DMP Melbourne) were 3 min. The total estimated time taken for human assessor (man-days) = total time taken per image (minutes) × number of retinal images/24/6.5. One man-day is equivalent to 6.5 h/day. For DLS, we recorded the time taken to pre-process and analyze the retinal images using a graphic processing unit (GPU) for 8 datasets. Each retinal image required 0.4 seconds.

### Statistical analysis

First, we calculated the overall area AUC of DLS and level of agreement of DLS in detection of 3 outcomes: any DR, referable DR and VTDR, with reference to human assessors. Level of agreement was assessed using Kappa coefficient: 0–0.2: slight agreement; 0.2–0.4: fair; 0.4–0.6: moderate; 0.6–0.8: good and; 0.8–1.0: excellent. Second, we analyzed the prevalence for any DR, referable DR and VTDR and time taken between the DLS and human assessors. Third, we performed a pooled analysis and used random-effect multivariate logistic regressions across 8 individual datasets on the risk factors for DLS and human-assessed DR outcomes. Then, the strength of the relationship with risk factors, assessed by odds ratios (OR) estimated from the meta-analysis, were compared between DLS and human assessors for statistical difference using Student’s *t*-tests and forest plots.^52^ Fourth, we calculated the AUC of the overall model to evaluate the discriminative ability of the combined risk factors for any DR, referable DR and VTDR as determined by DLS and human assessors. All data were expressed as mean (with standard deviation), number (with %) or standardized ORs (with 95% confidence intervals (CI)) with a *p*-value <0.05 considered to be statistically significant. All statistical analysis was performed using R Statistical Software (version 3.4.3; R Foundation for Statistical Computing, Vienna, Austria). With expected referable DR prevalence, DLS sensitivity and specificity of 5, 90, and 90%, respectively, the sample size required will be 7683 patients with desired precision of 0.03, 95% confidence interval.

## Supplementary information

Supplementary.

## Data Availability

The datasets used in this study originated from different principal investigators from different countries. Upon request, the corresponding authors, D.S.W.T and T.Y.W., can send the data request to the individual principal investigator to seek clearance from them.

## Supplementary information

**Supplementary information** accompanies the paper on the *npj Digital Medicine* website (10.1038/s41746-019-0097-x).

## Code availability

The AI system described in this study is kept at the Singapore Eye Research Institute (SERI) and National University of Singapore (NUS). The underlying algorithm is copyrighted by SERI, NUS and will not be available to public.
