# Supplementary material for: Deep learning in estimating prevalence and systemic risk factors for diabetic retinopathy: a multi-ethnic study
Source: NPJ Digit Med. 2019 Apr 10;2:24. doi: 10.1038/s41746-019-0097-x (PMC6550209; doi:10.1038/s41746-019-0097-x)
Supplement: Supplementary file 1 — Supplementary. [file 41746_2019_97_MOESM1_ESM.pdf]

Supplementary

Supplementary Figure 1: The breakdown of the patients of each dataset – Singapore Integrated Diabetic Retinopathy Program (SiDRP) 2014-15, Singapore Malay Eye Study (SIMES), Singapore Indian Eye Study (SINDI), Singapore Chinese Eye Study (SCES), Beijing Eye Study (BES), African American Eye Study (AFEDS), Chinese University of Hong Kong (CUHK) and Diabetes Management Project Melbourne (DMP)

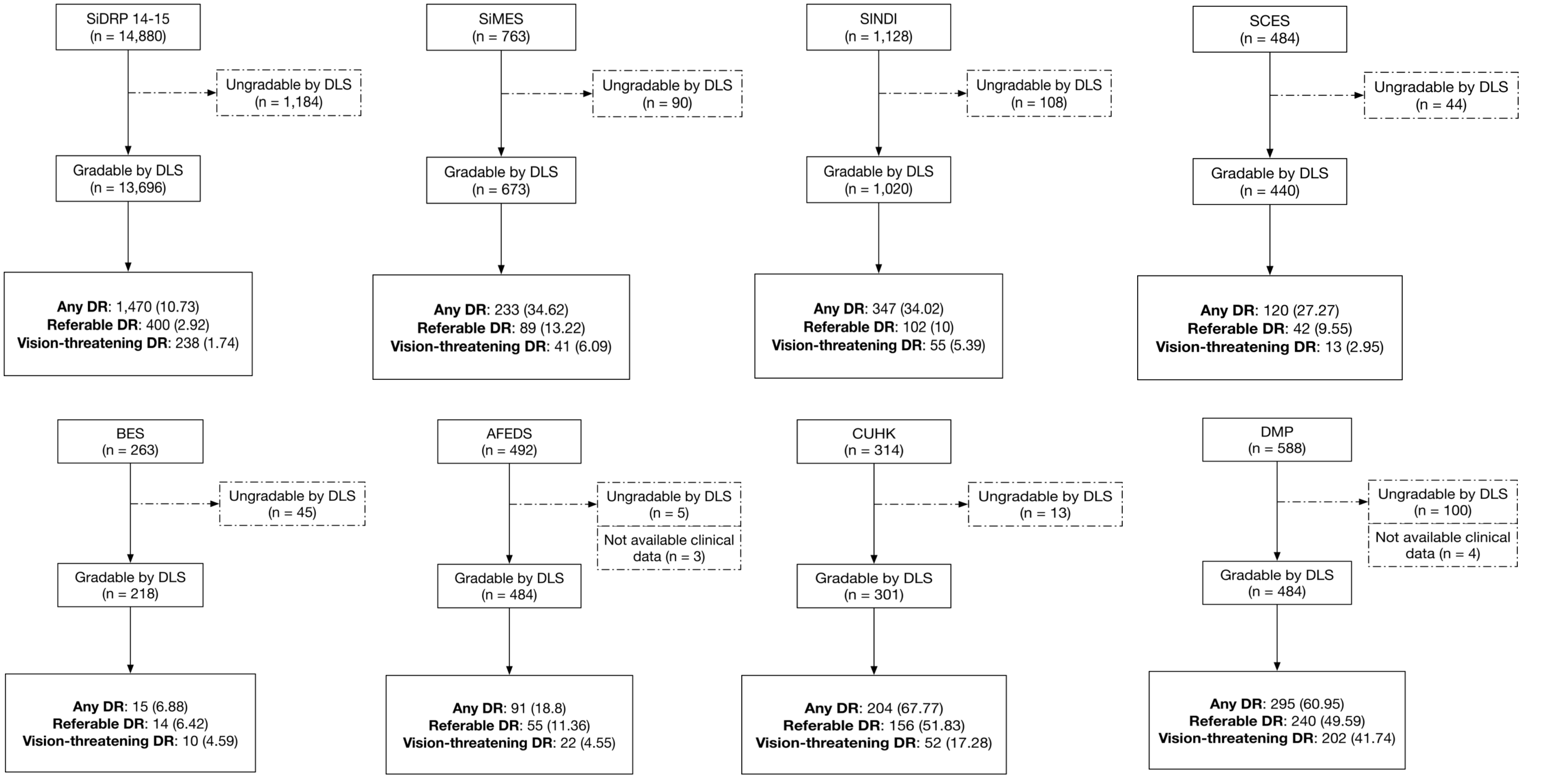

Supplementary Figure 2: The area under curve of combined systemic risk factors in discriminating any diabetic retinopathy (DR), referable DR and vision-threatening DR on the overall 8 multi-ethnic diabetic cohorts, analyzed using deep learning system vs human assessors. The risk factors including age, duration of diabetes, HbA1c, systolic and diastolic blood pressure, body mass index, total cholesterol and triglycerides. The 8 datasets consist of Singapore Integrated Diabetic Retinopathy Screening Program (SiDRP) between 2014 and 2015 (SiDRP 14-15), Singapore Malay Eye Study (SIMES), Singapore Indian Eye Study (SINDI), Singapore Chinese Eye Study (SCES), Beijing Eye Study (BES), African American Eye Study (AFEDS), Chinese University of Hong Kong (CUHK) and Diabetes Management Project Melbourne (DMP Melb).

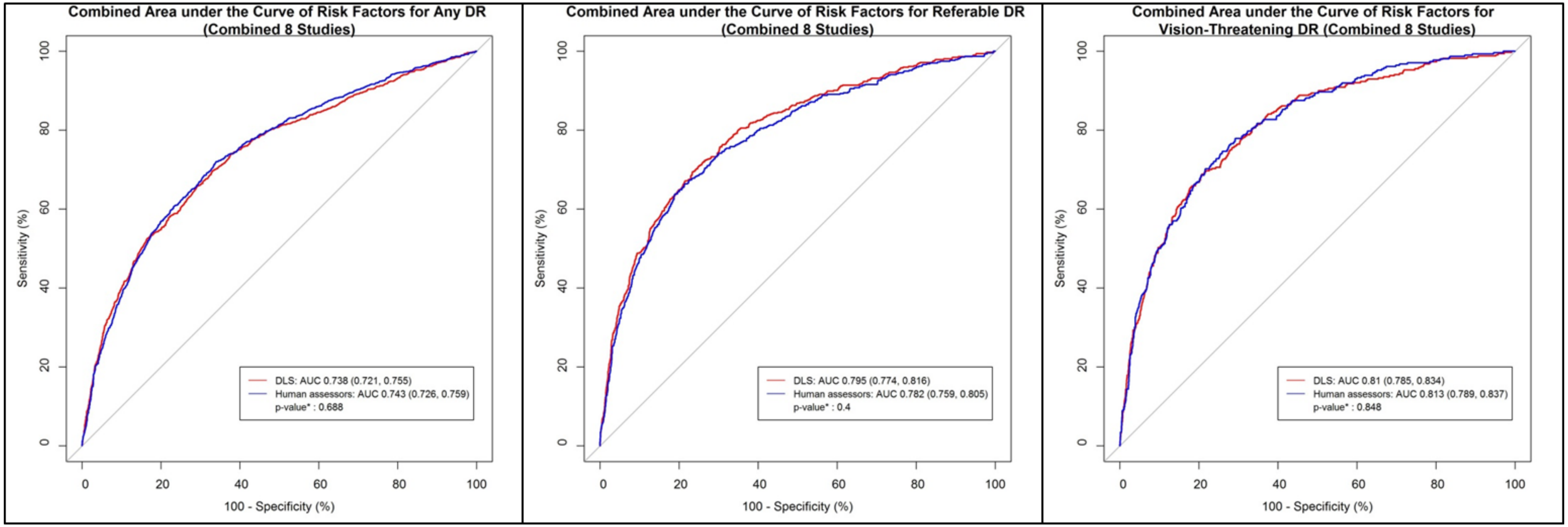

Any DR: defined as mild non-proliferative DR (NPDR) or worse
Referable DR: defined as moderate NPDR or worse
Vision-threatening DR: defined as severe NPDR, proliferative DR
The 95% CIs for each respective AUC were computed with stratified bootstrap replicates for the combined dataset from pooling all participants,
P-value between DLS and human assessors' ROC curves is computed using DeLong et al. (1988) method for paired ROC curves

Supplementary Figure 3a: The area under curve of combined systemic risk factors in discriminating any diabetic retinopathy (DR), analyzed using deep learning system vs human assessors on the Singapore Integrated Diabetic Retinopathy Screening Program (SiDRP) between 2014 and 2015 (SiDRP 14-15), Singapore Malay Eye Study (SIMES), Singapore Indian Eye Study (SINDI), Singapore Chinese Eye Study (SCES), Beijing Eye Study (BES), African American Eye Study (AFEDS), Chinese University of Hong Kong (CUHK) and Diabetes Management Project Melbourne (DMP Melb).

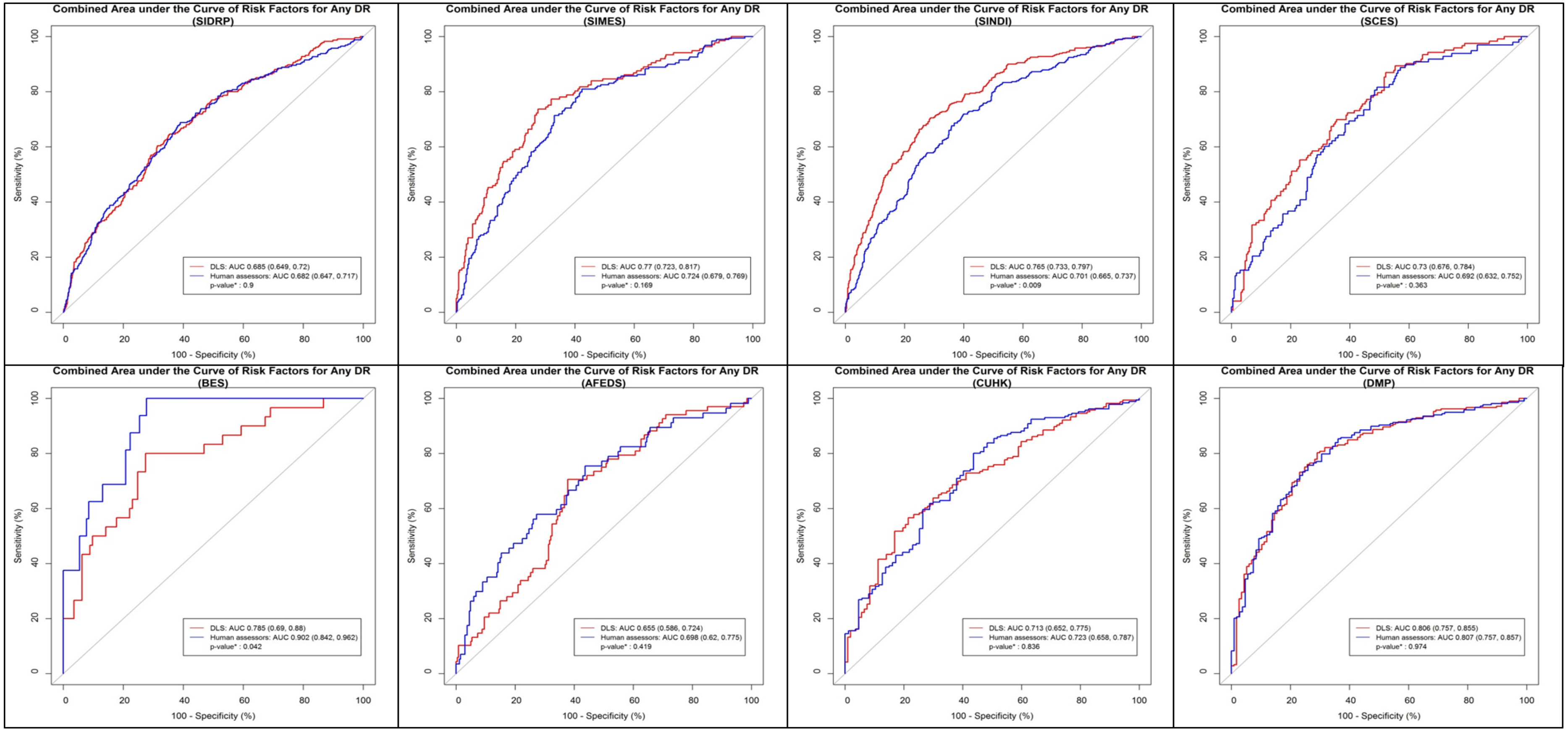

Risk factors include age, duration of diabetes, HbA1c, systolic and diastolic blood pressure, body mass index, total cholesterol and triglycerides.
The 95% CIs for each respective AUC were computed with stratified bootstrap replicates for each respective dataset,
P-value between DLS and human assessors' ROC curves is computed using DeLong *et al.* (1988) method for paired ROC curves

Supplementary 3b: The area under curve of combined systemic risk factors in discriminating referable DR, analyzed using deep learning system vs human assessors on the 8 datasets.

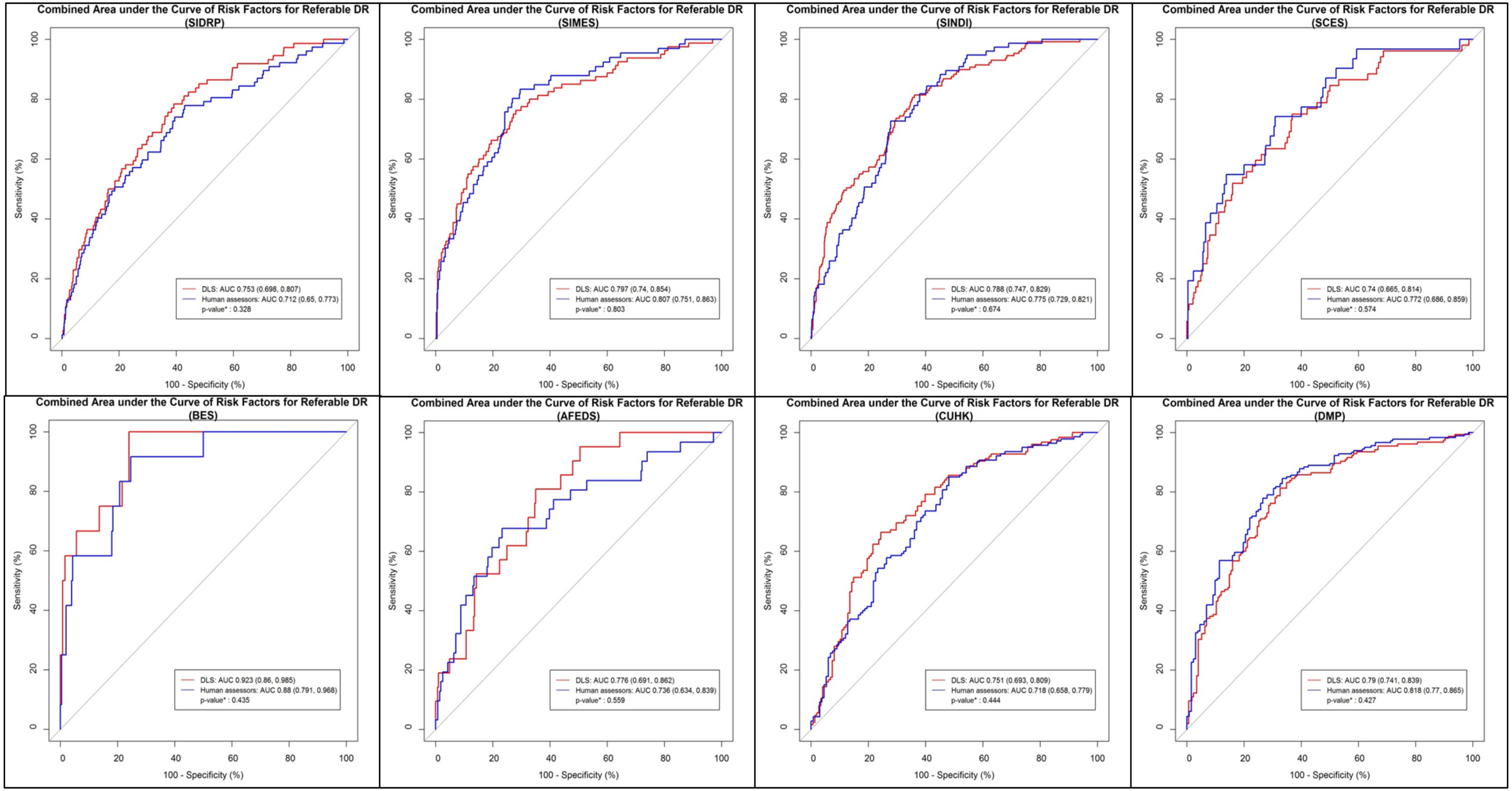

Risk factors include age, duration of diabetes, HbA1c, systolic and diastolic blood pressure, body mass index, total cholesterol and triglycerides.
The 95% CIs for each respective AUC were computed with stratified bootstrap replicates for each respective dataset,
P-value between DLS and human assessors' ROC curves is computed using DeLong *et al.* (1988) method for paired ROC curves

Supplementary 3c: The area under curve of combined systemic risk factors in discriminating vision-threatening DR (VTDR), analyzed using deep learning system vs human assessors on the 8 datasets.

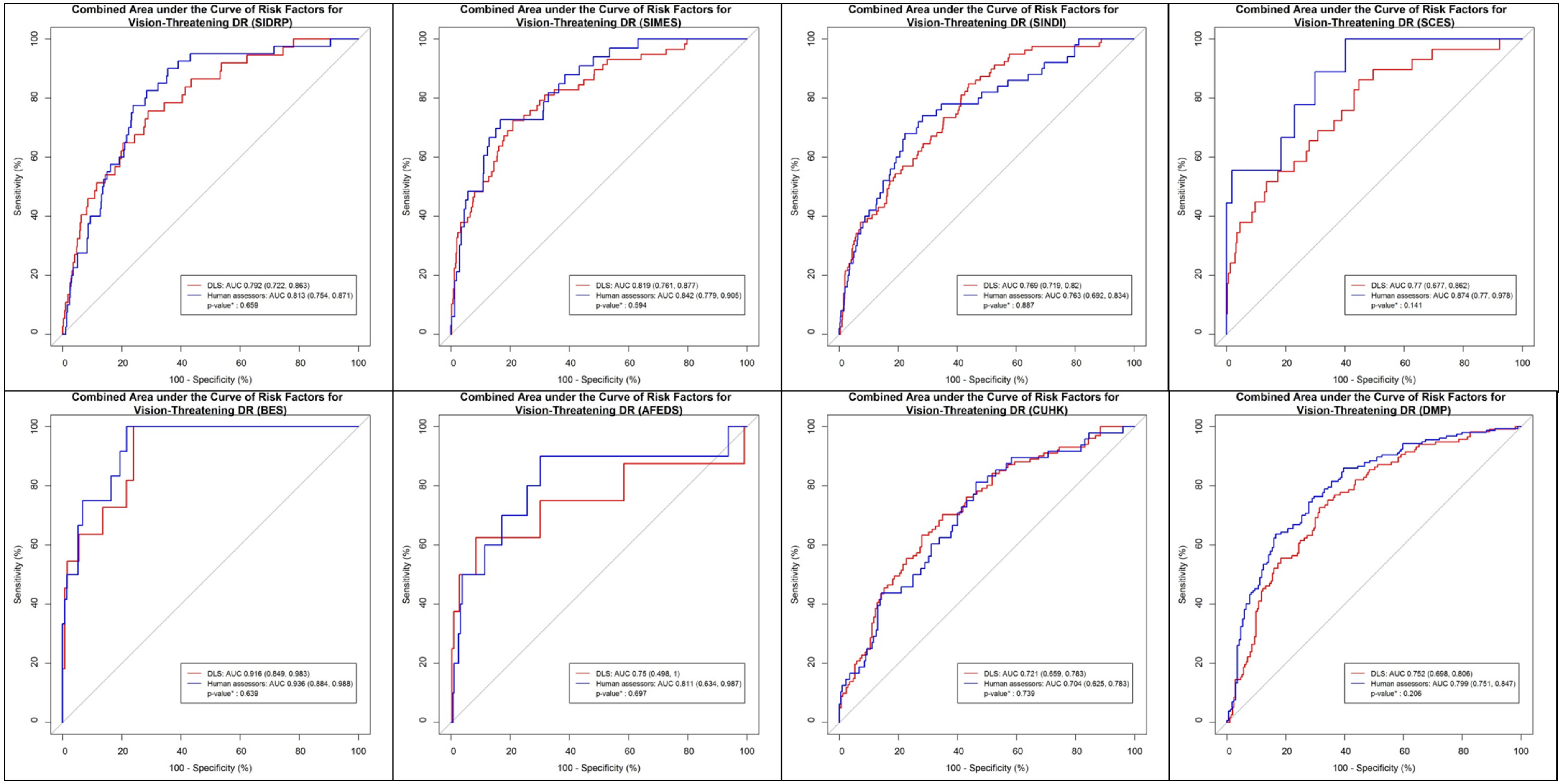

Risk factors include age, duration of diabetes, HbA1c, systolic and diastolic blood pressure, body mass index, total cholesterol and triglycerides.
The 95% CIs for each respective AUC were computed with stratified bootstrap replicates for each respective dataset,
P-value between DLS and human assessors' ROC curves is computed using DeLong *et al.* (1988) method for paired ROC curves

Supplementary Table 1: The total number and time taken of retinal images analyzed by a deep learning system (DLS) and a human assessor on the 8 different datasets.

	SiDRP 14-15	SiMES	SINDI	SCES	BES	AFEDS	CUHK	DMP Melb
**Patients' demographics and vascular risk factors**								
Total number of retinal images (patients)	68,286 (14,880)	3,952 (763)	6,329 (1,128)	5,284 (484)	429 (263)	3,383 (492)	2,199 (314)	3,431 (588)
Total number of retinal images (patients) deemed gradable by DLS	62,941 (13,696)	3,515 (673)	5,803 (1020)	4,925 (440)	378 (218)	3,359 (484)	2,131 (314)	2,850 (588)
Ungradable retinal images (patients)	5,345 (1,184)	437 (90)	526 (108)	359 (44)	51 (45)	24 (8)	68 (13)	581 (588)
**Reference Standard (Human Assessors)**	2 professional graders, 1 retinal specialist	1 professional grader, 1 retinal specialist	1 professional grader, 1 retinal specialist	1 professional grader, 1 retinal specialist	2 board-certified ophthalmologists	2 retinal specialists	2 retinal specialists	2 professiona[illegible] graders
**DLS#**								
i. Time taken to analyze all images (hours)	7.59	0.44	0.70	0.59	0.05	0.38	0.24	0.38
ii. Time taken to analyze all images (days)	0.32	0.02	0.03	0.02	0.00	0.02	0.01	0.02
iii. Additional time taken for secondary manual grading for DLS ungradable images (hours)	89.08	7.28	8.77	5.98	0.85	0.4	1.13	9.68
iv. Additional time taken for secondary manual grading for DLS ungradable images (man-days) *	13.71	1.12	1.35	0.92	0.13	0.06	0.17	1.49
Total time taken for DLS + manual grading by ungradable images (days)	14.03	1.14	1.38	0.94	0.13	0.08	0.18	1.51
Total time taken for DLS + manual grading by ungradable images (weeks)	2.81	0.23	0.28	0.19	0.03	0.02	0.04	0.30
**Human Assessor**								
i. Time taken to analyze 1 image (minutes)	2	3	3	3	3	3	5	3
ii. Time taken to analyze all images (hours)	2276.2	197.6	316.5	264.2	21.5	169.2	183.3	171.6
iii. Time taken to analyze all images (days)	350.2	30.4	48.7	40.6	3.3	26.0	28.2	26.4
iv. Time taken to analyze all images (weeks)	70.0	6.1	9.7	8.1	0.7	5.2	5.6	5.3

Singapore Integrated Diabetic Retinopathy Screening Program (SiDRP) between 2014 and 2015 (SiDRP 14-15), Singapore Malay Eye Study (SIMES), Singapore Indian Eye Study (SINDI), Singapore Chinese Eye Study (SCES), Beijing Eye Study (BES), African American Eye Study (AFEDS), Chinese University of Hong Kong (CUHK) and Diabetes Management Project Melbourne (DMP Melb).

#Each image requires 0.4 seconds to be analyzed by DLS

*1 man-day is equivalent to 6.5 hours/day; 5 working days are included in a working week. These tables did not include the annual/sick leave or public holidays

Supplementary Table 2: The multivariate analysis of systemic vascular risk factors with referable diabetic retinopathy (DR) (defined as moderate non-proliferative DR, severe non-proliferative DR, proliferative DR and diabetic macular edema) diagnosed by deep learning system, as compared to human assessors in Singapore Integrated Diabetic Retinopathy Screening Program (SiDRP) between 2014 and 2015 (SiDRP 14-15), Singapore Malay Eye Study (SIMES), Singapore Indian Eye Study (SINDI), Singapore Chinese Eye Study (SCES), Beijing Eye Study (BES), African American Eye Study (AFEDS), Chinese University of Hong Kong (CUHK) and Diabetes Management Project Melbourne (DMP Melb)

	**SiDRP 2014-15 (n=13,696)**					**SiMES (n= 673)**				
	**DLS (Standardized OR, 95% CI)**	**P value***	**Human assessors (Standardized OR, 95% CI)**	**P value***	**P value****	**DLS (Standardized OR, 95% CI)**	**P value***	**Human assessors (Standardized OR, 95% CI)**	**P value***	**P value****
Age (years)	0.69 (0.51, 0.94)	**0.018**	0.77 (0.57, 1.03)	0.082	0.625	0.68 (0.49, 0.95)	**0.025**	0.58 (0.4, 0.83)	**0.003**	0.526
Gender (Female)	0.59 (0.35, 1)	0.052	0.65 (0.39, 1.08)	0.102	0.801	1.37 (0.76, 2.51)	0.305	1.14 (0.6, 2.19)	0.691	0.685
Duration of diabetes (years)	1.26 (0.99, 1.57)	0.051	1.2 (0.95, 1.5)	0.115	0.796	1.76 (1.37, 2.26)	**<0.001**	1.48 (1.13, 1.93)	**0.004**	0.353
HbA1c (%)	1.46 (1.22, 1.72)	**<0.001**	1.42 (1.2, 1.67)	**<0.001**	0.85	1.86 (1.39, 2.5)	**<0.001**	1.84 (1.34, 2.53)	**<0.001**	0.964
Systolic Blood Pressure (mmHg)	1.58 (1.21, 2.07)	**0.001**	1.27 (0.97, 1.66)	0.078	0.264	3.19 (2.13, 4.88)	**<0.001**	3.8 (2.45, 6.06)	**<0.001**	0.577
Diastolic Blood Pressure (mmHg)	1.03 (0.76, 1.39)	0.867	1.03 (0.77, 1.4)	0.825	0.971	0.57 (0.38, 0.83)	**0.005**	0.47 (0.3, 0.71)	**0.001**	0.513
Body Mass Index (kg/m^2)	0.69 (0.51, 0.92)	**0.014**	0.74 (0.55, 0.97)	**0.033**	0.766	0.9 (0.67, 1.2)	0.465	0.78 (0.56, 1.07)	0.124	0.519
Total Cholesterol (mmol/L)	1.38 (0.68, 2.65)	0.352	1.04 (0.5, 2.05)	0.903	0.578	0.78 (0.57, 1.06)	0.114	0.76 (0.54, 1.05)	0.102	0.908

Triglycerides (mmol/L)	0.76 (0.5, 1.13)	0.19	0.96 (0.65, 1.41)	0.845	0.417	1.01 (0.74, 1.35)	0.929	0.97 (0.69, 1.32)	0.846	0.839

	SINDI (n=1020)					SCES (n=440)				
	**DLS (Standardized OR, 95% CI)**	**P value***	**Human assessors (Standardized OR, 95% CI)**	**P value***	**P value****	**DLS (Standardized OR, 95% CI)**	**P value***	**Human assessors (Standardized OR, 95% CI)**	**P value***	**P value****
Age (years)	0.71 (0.53, 0.95)	**0.02**	0.75 (0.53, 1.06)	0.105	0.813	0.63 (0.41, 0.94)	**0.027**	0.42 (0.24, 0.72)	**0.002**	0.252
Gender (Female)	0.72 (0.46, 1.13)	0.154	0.72 (0.41, 1.24)	0.243	0.996	0.5 (0.24, 1.01)	0.058	0.55 (0.22, 1.35)	0.201	0.872
Duration of diabetes (years)	1.97 (1.6, 2.44)	**<0.001**	1.45 (1.13, 1.85)	**0.003**	0.059	1.45 (1.05, 1.98)	**0.021**	1.13 (0.72, 1.72)	0.573	0.37
HbA1c (%)	1.87 (1.5, 2.34)	**<0.001**	1.62 (1.27, 2.08)	**<0.001**	0.399	1.48 (1.08, 2.03)	**0.014**	1.65 (1.13, 2.42)	**0.009**	0.664
Systolic Blood Pressure (mmHg)	1.71 (1.29, 2.27)	**<0.001**	1.77 (1.26, 2.49)	**0.001**	0.871	1.78 (1.18, 2.73)	**0.007**	2.54 (1.51, 4.39)	**0.001**	0.308
Diastolic Blood Pressure (mmHg)	0.64 (0.47, 0.87)	**0.005**	0.56 (0.37, 0.81)	**0.003**	0.569	0.76 (0.47, 1.2)	0.245	0.54 (0.28, 0.99)	0.056	0.395
Body Mass Index (kg/m^2)	0.86 (0.68, 1.07)	0.202	0.73 (0.53, 0.97)	**0.035**	0.364	0.82 (0.58, 1.13)	0.228	0.88 (0.58, 1.3)	0.527	0.783
Total Cholesterol (mmol/L)	0.97 (0.76, 1.23)	0.796	1.2 (0.9, 1.59)	0.206	0.258	0.95 (0.66, 1.33)	0.764	1.06 (0.68, 1.61)	0.791	0.693
Triglycerides (mmol/L)	1.05 (0.84, 1.28)	0.65	0.85 (0.62, 1.12)	0.28	0.254	1.05 (0.74, 1.42)	0.79	1.27 (0.84, 1.82)	0.214	0.441

	BES (n= 218)	AFEDS (n=484)

	DLS (Standardized OR, 95% CI)	P value*	Human assessors (Standardized OR, 95% CI)	P value*	P value**	DLS (Standardized OR, 95% CI)	P value*	Human assessors (Standardized OR, 95% CI)	P value*	P value**
Age (years)	2.51 (0.89, 8.88)	0.105	0.78 (0.31, 1.92)	0.585	0.11	0.8 (0.43, 1.48)	0.474	0.7 (0.42, 1.16)	0.163	0.733
Gender (Female)	5.44 (0.78, 61.82)	0.121	1 (0.23, 4.82)	0.996	0.205	1.22 (0.44, 3.55)	0.707	1.09 (0.46, 2.71)	0.851	0.87
Duration of diabetes (years)	3.58 (1.36, 12)	**0.018**	3.18 (1.45, 7.81)	**0.006**	0.861	1.55 (1.02, 2.34)	**0.036**	1.86 (1.28, 2.71)	**0.001**	0.528
HbA1c (%)	2.7 (1.47, 5.91)	**0.004**	2.48 (1.53, 4.38)	**0.001**	0.842	1.89 (1.27, 2.82)	**0.002**	1.53 (1.07, 2.15)	**0.016**	0.427
Systolic Blood Pressure (mmHg)	1.58 (0.58, 4.58)	0.371	2.04 (0.86, 5.09)	0.109	0.705	1.32 (0.71, 2.38)	0.375	1.31 (0.79, 2.15)	0.291	0.991
Diastolic Blood Pressure (mmHg)	0.84 (0.25, 2.89)	0.777	0.7 (0.24, 2.05)	0.516	0.829	0.43 (0.2, 0.84)	**0.017**	0.53 (0.29, 0.94)	**0.033**	0.626
Body Mass Index (kg/m^2)	1.14 (0.42, 2.98)	0.778	1.46 (0.64, 3.13)	0.343	0.692	0.84 (0.5, 1.32)	0.469	0.92 (0.61, 1.34)	0.663	0.774
Total Cholesterol (mmol/L)	0.48 (0.16, 1.29)	0.146	0.92 (0.43, 2.04)	0.828	0.319	1.19 (0.67, 2.07)	0.54	1.24 (0.78, 1.97)	0.355	0.907
Triglycerides (mmol/L)	0.27 (0.05, 0.89)	0.069	1.18 (0.57, 2.12)	0.615	0.064	0.72 (0.36, 1.27)	0.305	0.82 (0.49, 1.28)	0.424	0.739

	CUHK (n= 301)					DMP Melbourne (n= 484)				
	**DLS (Standardized OR, 95% CI)**	**P value***	**Human assessors (Standardized OR, 95% CI)**	**P value***	**P value****	**DLS (Standardized OR, 95% CI)**	**P value***	**Human assessors (Standardized OR, 95% CI)**	**P value***	**P value****

Age (years)	0.56 (0.4, 0.77)	**0.001**	0.63 (0.45, 0.86)	**0.005**	0.61	0.6 (0.44, 0.81)	**0.001**	0.64 (0.46, 0.87)	**0.006**	0.788
Gender (Female)	2.14 (1.26, 3.66)	**0.005**	1.75 (1.04, 2.95)	**0.035**	0.597	0.34 (0.19, 0.6)	**<0.001**	0.3 (0.16, 0.55)	**<0.001**	0.796
Duration of diabetes (years)	0.72 (0.52, 0.96)	**0.031**	0.77 (0.57, 1.02)	0.076	0.738	1.59 (1.22, 2.12)	**0.001**	1.84 (1.35, 2.56)	**<0.001**	0.51
HbA1c (%)	1.7 (1.24, 2.4)	**0.002**	1.81 (1.31, 2.59)	**0.001**	0.79	1.94 (1.46, 2.63)	**<0.001**	2.42 (1.74, 3.49)	**<0.001**	0.339
Systolic Blood Pressure (mmHg)	1.74 (1.25, 2.45)	**0.001**	1.55 (1.13, 2.17)	**0.008**	0.635	1.28 (0.94, 1.78)	0.125	1.47 (1.04, 2.1)	**0.032**	0.585
Diastolic Blood Pressure (mmHg)	0.85 (0.61, 1.19)	0.356	0.85 (0.61, 1.18)	0.334	0.992	0.83 (0.59, 1.15)	0.26	0.74 (0.51, 1.05)	0.098	0.646
Body Mass Index (kg/m^2)	0.86 (0.64, 1.14)	0.299	0.95 (0.72, 1.25)	0.725	0.609	1.67 (1.19, 2.38)	**0.004**	1.58 (1.1, 2.29)	**0.014**	0.825
Total Cholesterol (mmol/L)	1.02 (0.78, 1.34)	0.88	1.06 (0.81, 1.4)	0.65	0.834	0.93 (0.7, 1.24)	0.622	0.76 (0.55, 1.04)	0.09	0.357
Triglycerides (mmol/L)	N/A		N/A			0.84 (0.63, 1.11)	0.222	0.88 (0.64, 1.22)	0.473	0.803

*P value is generated by logistic regression analysis

** P value for the difference in standardized Odd Ratios in the multivariate regression between deep learning system and human assessors, generated using student T-test (2-Tailed)

Referable diabetic retinopathy (DR): defined as moderate non-proliferative DR, severe non-proliferative DR, proliferative DR

Vision-threatening diabetic retinopathy (DR): defined as severe non-proliferative DR, proliferative DR
